# Alcohol Consumption and Cigarette Smoking among Young Adults: An Instrumental Variable Analysis Using Alcohol Flushing

**DOI:** 10.3390/ijerph182111392

**Published:** 2021-10-29

**Authors:** Yongho Jee, Susan Park, Eunu Yuk, Sung-il Cho

**Affiliations:** 1 Advanced Biomedical Research Institute, Ewha Womans University Seoul Hospital, 260, Gonghang-daero, Gangseo-gu, Seoul 07804, Korea; jyongho@ewha.ac.kr; 2 Institute for Community Care and Health Equity, Chung-Ang University, 84 Heukseok-ro, Dongjak-gu, Seoul 06974, Korea; lovelysu99@hotmail.com; 3 Korea Health Promotion Institute, Namsan Square Building, Toegyero 173, Jung-gu, Seoul 04554, Korea; eunu.yuk@gmail.com; 4 Department of Public Health Science, Graduate School of Public Health, Seoul National University, 1 Gwanak-ro, Gwanak-gu, Seoul 08826, Korea

**Keywords:** alcohol drinking, smoking status, alcohol flushing status, instrument variable, young adults

## Abstract

Association between drinking and smoking has remained controversial since the association between two studies were influenced by various confounding. Thus, our study aimed to explore the causal effect of alcohol consumption and cigarette smoking using alcohol flushing as an instrument variable, which is free from confounders. We analyzed cross-sectional survey data from 2500 Korean young adults (1600 men and 900 women). Alcohol flushing was strongly associated with log transformed alcohol consumption (F = 272). In men, alcohol non-flushers were 1.41 times (95% CI 1.28–1.55) more likely to smoke 100 cigarettes in their lifetime in logistic regression analysis. Alcohol non-flushers were also 1.3 times (95% CI 1.21–1.40) more likely to become daily smokers and 1.39 times (95% CI 1.27–1.51) more likely to be current smokers than alcohol flushers. However, in an IV analysis, no causal relationships between alcohol consumption and smoking status were found. Alcohol consumption, on the other hand, was causally associated with lowering nicotine dependence and former smoking in men. Alcohol consumption determined by alcohol flushing status does not appear to be causally linked to the smoking behavior of young adults. The relationship between alcohol consumption and nicotine dependence and smoking cessation needs further study.

## 1. Introduction

In the 1980s, the prevalence of smoking for Korean males was estimated as close to 80% [1]. This high smoking prevalence continued until the 1990s, began to steadily decline from 1998 until 2013, and has since stagnated at the 40% level.

However, the decline in smoking prevalence has not been even across the male population. For example, the prevalence of smoking for young adult men aged 20–29 and 30–39 was estimated in 2016 to be over 40% and 50%, respectively. As a result, smoking in young adult populations is still regarded as a serious social problem [2]. In addition, the prevalence of alcohol consumption among young adults is far above the smoking prevalence, remaining over 70% for young adult males [2].

In this context, smoking and drinking have long been viewed as co-existing unhealthy lifestyle habits among young adults. When continued into the long term, as is common for addictive behaviors, smoking and drinking may lead to various chronic diseases manifesting in middle to old age, underscoring the importance of targeting interventions towards young adults [3]. Interestingly, previous studies have reported an interaction between smoking and alcohol drinking with respect to the onset of esophageal and stomach cancer [4,5,6]. It may therefore be possible that the negative impact of alcohol or smoking behaviors individually is exacerbated when both occur simultaneously, and further, that one behavior serves as a risk factor for the other. This latter possibility is supported by a recent study by Masaoka et al. [7] suggesting alcohol to be a determinant of smoking status and this is further supported by epidemiological studies showing a strong association between the genetic variant ALDH2 (implicated in development of the alcohol flushing reaction) and smoking cessation [7]. However, whilst such findings are promising, there remains a lack of research examining the extent to which the consumption of alcohol is causally relevant with respect to smoking. 

Identifying a causal relationship between drinking and smoking is both clinically and politically significant, as it can provide a clear indication of the most effective targets for developing and implementing health interventions. It may therefore be possible to focus on an upstream risk factor, with an emphasis upon prevention as opposed to treatment.

One approach to assessing the causal relationship between alcohol consumption and smoking is to adopt an instrumental variable (IV) approach. Such approaches use variables as instruments to control for unmeasured confounding and allow for unbiased causal effect estimates in cases where an IV is associated with the endogenous explanatory variable of interest, independent of confounders of the explanatory and outcome variable, and independent with respect to the outcome of interest [8]. An IV satisfying these assumptions is considered to be valid. For example, Mendelian randomization (MR) studies using genetic variants as IVs [9] have provided a substantial body of evidence regarding the health impact of alcohol consumption [10,11,12,13,14,15]. Another method for inferring causality is causal mediation, which assumes that there is a mediator between exposure and outcome. However, in our study, we used the IV analysis methodology because it was assumed that there was no separate mediator between drinking and smoking. In East Asia, the acetaldehyde dehydrogenase 2 (ALDH2) gene, responsible for the oxidation of acetaldehyde to acetate, has been one popular candidate as an IV for MR studies [15,16], though few of these studies have centered on young adults. ALDH2 gene response is also called Asian flush or Asian glow since this phenomenon is associated with inherited deficiency in the ALDH2 enzyme among East Asians [16].

Since Yokoyama and colleagues (2003) developed an alcohol flushing questionnaire as an alternative method to identify ALDH2-deficient individuals, studies by Hsiao et al. (2019) and Shin et al. (2018) reported the validity of the alcohol flushing questionnaire [17,18]. Validity of alcohol flushing was found to be highly reliable as an instrumental variable from previous studies detecting subjects with inactive ALDH2 in the East Asian population [19,20,21]. In this study, we aimed to examine the validity of alcohol flushing, recorded using questionnaire as an IV, and investigate the causal effect of alcohol consumption on cigarette smoking specifically within the Korean young adult population.

## 2. Materials and Methods

### 2.1. Study Setting and Design

Our study analyzed cross-sectional questionnaire data obtained from Korean adults aged 20–29, containing information on smoking, alcohol consumption, and potential related factors. Two thousand five hundred young adults were proportionally sampled with respect to sex, age, region, and smoking status among panels from Embrain. Korean analytics and advisory company Embrain has managed 13 million panels since 1998. Panels who were interested in our survey voluntarily participated in September 2017. All participants gave informed consent for current study. Among the 2500 young adult participants, 120 non-drinkers were excluded, with 2380 young adults (men, 1518; women, 862) remaining in the following analyses. The survey was conducted online for those who volunteered to participate, using questionnaires approved by Seoul National University Institutional Review Board (IRB) committee (IRB No. 1704/001-016). 

### 2.2. Measurement of Variables

A range of variables are considered in our study. All questionnaires were recorded by self-report from voluntarily participants. For alcohol consumption, weekly alcohol drinking frequency and alcohol drinking amount per session was recorded. Smoking and alcohol drinking variables were selected based on the variables in Korea national health and nutrition examination survey (KNHANES) or Korea Youth Risk Behavior Web-based Survey (KYRBS) [22,23]. Alcohol amount was calculated as an average using weekly alcohol drinking frequency and alcohol drinking amount per time, with volume and concentration of alcohol. Regarding smoking behaviors, smoking amount, smoking duration, nicotine dependency, and smoking inhalation were utilized. Nicotine dependency was measured using the Fragerstrom test [24], and participants with a score higher than 5 were defined as highly addicted smokers. Finally, information on residence, occupation, education, and income were also measured as potential confounders in estimating the association between alcohol drinking and smoking. 

### 2.3. Alcohol Flushing

For alcohol flushing, participants were asked to answer following two questionnaires. The first question was “Do you usually get facial flushing even with a small amount of alcohol drinking?” (a) Yes, soon after first mouthful; (b) Yes, after drinking a small amount of alcohol; (c) Yes, but only after drinking large amount of alcohol; or (d) No. The second question was “After how many drinks do you usually experience hot flushes or dizziness? (a) 1 glass, (b) 2 glasses, (c) 3 glasses, (d) 4 glasses, (e) 5 glasses or more, or (f) None.”

### 2.4. Statistical Analysis

**Figure 1:**
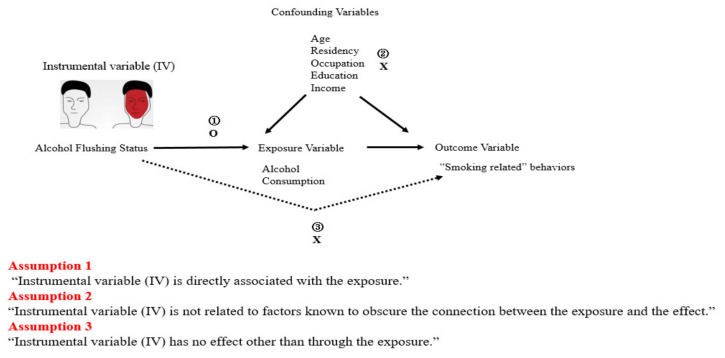
A directed acyclic graph depicting the assumed associations for unbiased inference using IV analysis. Dashed lines represent associations required to be zero in order for assumptions 2 and 3 to be satisfied.

IV analysis requires three assumptions. Initially, we aimed to examine whether alcohol flushing status can be used as a valid IV in estimating the effect of alcohol consumption upon smoking. In relation to the first IV assumption, we assessed the extent to which alcohol flushing is associated with alcohol consumption. The association between alcohol flushing status, smoking status, and possible confounders of alcohol consumption and smoking was estimated using logistic regression models (Figure 1). 

Finally, we conducted an IV analysis by using alcohol flushing status as an IV to assess the causal relationship between amount of alcohol consumed and smoking. In this study, we utilized the alcohol consumption as continuous variable for 10 g alcohol consumed per week. Association estimates are presented on the odds ratio scale. All statistical analyses were conducted with SAS version 9.3 (SAS Institute, Cary, NC, USA) and Stata 14.0 (StataCorp., College Station, TX, USA)

## 3. Results

### 3.1. Description of the Study Population

**Figure 2:**
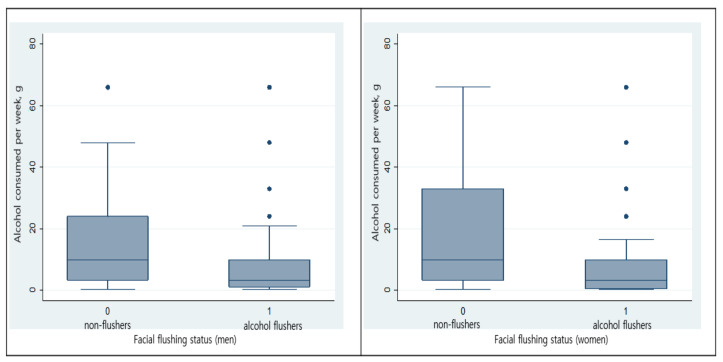
Facial flushing status in men (**left side**) and women (**right side**), with 0 representing alcohol non-flushers and 1 representing alcohol flushers.

Table 1 presents the study characteristics according to alcohol flushing status for men and women. Alcohol flushers accounted for 32.5% and 33.6% of men and women, respectively. In both the male and female subgroups, no meaningful differences were found between alcohol flushers and non-flushers in terms of age, residency, occupation, education, or income. Considering the smoking related variables, there was no substantial difference in lifetime smoking status, having smoked 100 cigarettes during lifetime, and daily smoking behavior by alcohol flushing status in men and women. There did, however, appear to be a difference by alcohol flushing group for males with a history of former smoking and nicotine dependency in men. There does appear to be a meaningful difference in amount of alcohol consumed per week between alcohol flushers and non-alcohol flushers for both men and women, as shown in Figure 2.

### 3.2. Alcohol Consumption and Smoking Related Variables: Association or Causal?

Table 2 shows the results from performing logistic regression and IV analyses using alcohol flushing as an IV in men. In the logistic regression analyses, the absence of alcohol flushing symptoms was positively associated with a range of smoking behaviors. Alcohol non-flushers were 1.96 times (95% confidence interval 1.56–2.45) more likely to smoke one puff of a cigarette in their lifetime and 1.67 times (95% CI 1.43–1.95) more likely to smoke one cigarette in their lifetime. However, alcohol flushing was not associated with having a history of quitting smoking. 

Table 2 also displays the results from performing an IV analysis using alcohol flushing as an IV for estimating the effect of alcohol drinking on smoking habits in men. There appeared to be no evidence of other causal relationships with respect to the smoking related variables. However, in this case participants consuming greater amounts of alcohol were more likely to be former smokers (OR 2.04, 95% CI 1.02–4.04), and increased alcohol intake was found to reduce nicotine dependence (OR 0.65, 95% CI 0.47–0.91). 

Table 3 gives the results of performing the logistic regression and IV analyses above with the subset of women. As with men, the absence of alcohol flushing symptoms was associated with a majority of smoking behaviors; Alcohol non-flushers were more likely to smoke one puff of a cigarette in their lifetime (OR 2.44, 95% CI 1.89–3.15) and, more likely to smoke one cigarette in their lifetime (OR 1.95, 95% CI = 1.64–2.32). However, no causal relationship was found with other smoking related variables when performing the IV analysis.

## 4. Discussion

Our present study aimed to examine the causal association between alcohol drinking and tobacco smoking in young adults utilizing observational and IV approaches. First, a strong association was detected between alcohol consumption and various smoking variables, such as lifetime smoking status, current smoking status, and nicotine dependency. However, the amount of alcohol consumed was not found to be causally associated with smoking status using alcohol flushing within an IV framework in men and women. Exceptionally, alcohol consumption, on the other hand, was causally associated with former smoking and lowering nicotine dependence in men.

Alcohol flushing has been used in various studies as an IV for alcohol consumption [12,15,19,21]. Yun et al. examined the validity of alcohol flushing, reporting a causal association of alcohol drinking upon coronary artery calcification [25]. However, studies specifically targeting young adults and using alcohol flushing are relatively rare. 

We found strong evidence of an association between alcohol drinking and alcohol flushing symptoms, with alcohol non-flushers consuming approximately twice as much alcohol in comparison to alcohol flushers. We also found no evidence of association between alcohol flushing status and potential confounders of alcohol consumption and smoking; specifically social economic status measured using residency, occupation, education and income. The strong association between alcohol flushing status and alcohol consumption, coupled with the absence of associations between alcohol flushing and potential confounders of alcohol and smoking, therefore supports the validity of alcohol flushing status as an IV [12,13,19,21].

Several studies have investigated the association between tobacco and alcohol use [26,27]. Whilst not finding smoking to be causally associated with alcohol drinking, there appears to be a consensus that many lifestyle behaviors are connected with smoking lifestyle behaviors. Our findings agree with this perspective, with alcohol drinking being unlikely to serve as cause of smoking. Targeting alcohol cessation as an intervention would therefore only have a limited impact on smoking cessation.

If the converse is true, and smoking is a cause of alcohol drinking, it could act as the underlying mechanism behind the two co-existing lifestyles. This provides the motivation for further research. In terms of alternative mechanisms driving the co-existence of alcohol and smoking behaviors, several explanations have emerged from recent studies. One possibility is that some individuals who have a physiological predisposition to using addictive drugs, such as alcohol and tobacco, are more likely to become dual users who use various drugs at the same time. However, previous studies failed to identify this addiction mechanism [28].

Another explanation is that alcohol drinking and tobacco smoking could be determined by confounding variables. This explanation agrees with the findings of our study though there is substantial debate as to whether there is a direct causal link between the two behaviors. For example, Taylor and his colleagues suggested that heaviness of smoking does not causally influence level of alcohol consumption [29]. Social and economic factors could potentially serve as such causal determinants, particularly as the association between social smoking and alcohol drinking has been reported by numerous studies [30,31].

As highlighted above, improving health care provision for young adults is a meaningful issue. Young adult smoking is one of the most important issues with respect to public health since it can be linked to a range of negative outcomes, such as hypertension, high cholesterol, and diabetes, only presenting in later life. Therefore, clarifying and targeting the risk factors of smoking in young adult population should be a prioritized. With these issues in mind, many studies have previously reported family smoking, social norms, peer smoking, and alcohol drinking as main risk factors with respect to smoking initiation and smoking status; however, due to the lack of data accessibility, a systematic research analyzing the causal effect among these risk factors was not reported [32,33,34]. In the context of this research and contemporary health issue, our study provides a major contribution by investigating the potential the association between drinking and smoking in the young adult population using an IV approach.

Future work will focus upon replication of the findings of this study using several genetic variants, (specifically rs671 in ALDH2 and rs1229984 in ADH1B) as IVs within an MR study design. Flushing syndrome is caused by a specific SNP: rs671. However, this SNP has also been associated with smoking cessation [35]. This might be a violation of one of the assumptions of an instrumental variable (Z affects the outcome Y only through X).

Our study tried a novel attempt compared to previous studies in the following attempts. Regarding the methodology, in our knowledge, our study is the first study that used alcohol flushing as an instrumental variable to clarify the association with tobacco smoking. In terms of our study population, we focused on young adults who were not treated with interest in other studies since they showed relatively lower risk of health outcomes associated with alcohol and smoking. Since those two modifiable life factors are associated with numerous risk of chronic disease, results from our study may be used as a basic evidence for policy makers who aim to plan regulation or management for those lifestyles.

Our study has several limitations that must be considered when interpreting our results. Since we used self-reported alcohol flushing variable, we were not able to guarantee the validity of alcohol flushing compared to flushing status defined by genetic examination. Unfortunately, we cannot assess whether the responses recorded for alcohol flushing accurately reflect the shortage of the alcohol decomposing enzyme responsible using the data provided alone though the use of genetic variants can serve as a more accurate measure [36]. In this study, alcohol flushing was strongly associated with log transformed alcohol consumption (F = 272), with alcohol flushing status explaining approximately 10.2% of alcohol consumption. Therefore, power might be adequate for this study. However, we feel this issue deserves more attention considering the limited sample size. Especially considering how large the confidence intervals are, “deeply inhale tobacco”, “former smoker”, and “social smokers” show similar OR compared to the linear regression for both men and women but with very large confidential intervals. Therefore, we feel the conclusions of this study should be interpreted with cautions.

## 5. Conclusions

Our study has showed that whilst alcohol drinking is closely related to smoking using ordinary alcohol consumption variables, causal association was not found using alcohol flushing as an IV among Korean young adults. However, our study showed that alcohol flushing can be used as an IV that can estimate alcohol consumption free from confounding variables. Further studies with using genetic variants that can improve the validity of IV are warranted in order to verify the causal association between alcohol consumption and tobacco smoking.

## Figures and Tables

**Table 1:** Characteristics of study subjects according to gender and alcohol flushing status.

		Alcohol Flushing in Men			Alcohol Flushing in Women		
		Flushers (*n* = 493)	Non-Flushers (*n* = 1025)	*p* Value	Flushers (*n* = 290)	Non-Flushers (*n* = 572)	*p* Value
Age, y	Mean (SD)	25.1 (2.6)	25.3 (2.5)	0.1595	25.4 (2.6)	25.4 (2.5)	0.4400
Residency	Rural (%)	36 (7.3)	39 (7.9)	0.6814	21 (7.2)	28 (4.9)	0.1598
Occupation	Professionals	152 (30.8)	277 (27.0)	0.1950	64 (22.2)	127 (22.2)	0.3583
	Clerical and sales	282 (57.2)	572 (55.8)		224 (77.3)	433 (75.7)	
	Others	59 (12.0)	176 (17.2)		1 (0.5)	12 (2.1)	
Education	<College	72 (14.6)	146 (14.2)	0.8621	36 (12.5)	92 (16.1)	0.1980
	≥College	421 (85.4)	879 (85.8)		254 (87.5)	480 (83.9)	
Income, ×10,000 won	<200	55 (11.1)	51 (10.3)	0.8813	28 (9.5)	54 (9.5)	0.6771
	200–599	317 (64.4)	320 (64.9)		161 (55.5)	335 (58.6)	
	≥600	121 (24.5)	122 (24.8)		102 (35.0)	182 (31.9)	
Smoking related variables							
Smoke one puff of cigarette in lifetime		430 (87.2)	916 (89.4)	0.2170	227 (78.3)	452 (79.0)	0.8005
Smoke one cigarette in lifetime		407 (82.6)	852 (83.1)	0.7836	204 (70.3)	406 (71.0)	0.8466
Smoke 100 cigarettes in lifetime		339 (68.8)	707 (69.0)	0.9331	157 (54.1)	305 (53.3)	0.8204
Daily smokers		256 (51.9)	513 (50.0)	0.3879	97 (33.5)	187 (32.7)	0.8234
Current smokers (daily or occasionally)		326 (66.1)	657 (64.1)	0.4386	144 (49.7)	279 (48.8)	0.8073
Quitting smoking		19 (3.83)	72 (7.07)	0.0394	24 (8.3)	49 (8.5)	0.9287
High nicotine dependence		132 (26.80)	204 (19.91)	0.0106	55 (18.9)	86 (15.1)	0.2780
Deeply inhale tobacco		138 (27.90)	287 (28.02)	0.9664	57 (19.5)	136 (23.8)	0.2814

SD; standard deviation.

**Table 2:** Association between alcohol consumption and various smoking behaviors: logistic regression analysis and the instrumental variable (IV) analysis using alcohol flushing as an IV in men.

Smoking Related Variables		Logistic Regression, per 10 g/day			IV Analysis		
	Yes/No	OR	95% CI	*p* Value	OR	95% CI	*p* Value
Smoke one puff of cigarette in lifetime	1346/172	1.96	1.56–2.45	<0.0001	1.26	0.87–1.81	0.218
Smoke one cigarette in lifetime	1259/259	1.67	1.43–1.95	<0.0001	1.04	0.76–1.43	0.784
Smoke 100 cigarettes in life time	1046/472	1.41	1.28–1.55	<0.0001	1.01	0.78–1.30	0.933
Daily smokers	764/754	1.30	1.21–1.40	<0.0001	0.90	0.71–1.14	0.388
Current smokers (daily or occasionally)	983/535	1.39	1.27–1.51	<0.0001	0.91	0.71–1.16	0.439
Quitted smoking	63/983	0.83	0.69–1.00	0.0502	2.04	1.02–4.04	0.043
High nicotine dependence	237/828	1.17	1.09–1.27	<0.0001	0.65	0.47–0.91	0.011
Deeply inhale tobacco	297/767	1.10	1.02–1.89	0.011	1.01	0.74–1.37	0.966

Adjusted for age, residency, education, and income level. OR; odds ratio; CI; confidence interval.

**Table 3:** Association between alcohol consumption and various smoking behaviors: logistic regression analysis and the instrumental variable (IV) analysis using alcohol flushing as an IV in women.

Smoking Related Variables		Logistic Regression, per 10 g/day			IV Analysis		
	Yes/No	OR	95% CI	*p* Value	OR	95% CI	*p* Value
Smoke one puff of cigarette in lifetime	673/183	2.44	1.89– 3.15	<0.0001	1.04	0.78–1.38	0.801
Smoke one cigarette in lifetime	610/252	1.95	1.64–2.32	<0.0001	1.03	0.79–1.33	0.847
Smoke 100 cigarettes in life time	462/400	1.50	1.35–1.67	<0.0001	0.97	0.77–1.23	0.820
Daily smokers	284/578	1.41	1.29–1.53	<0.0001	0.97	0.76–1.24	0.824
Current smokers (daily or occasionally)	423/439	1.45	1.32–1.60	<0.0001	0.97	0.77–1.23	0.807
Quitted smoking	39/423	0.87	0.72–1.06	0.9894	1.03	0.57–1.84	0.929
High nicotine dependence	79/404	1.20	1.07–1.34	0.0019	0.79	0.52–1.21	0.279
Deeply inhale tobacco	108/375	1.17	1.05–1.30	0.0041	1.24	0.84–1.82	0.283

Adjusted for age, residency, education, and income level. OR; odds ratio; CI; confidence interval.

## Data Availability

Restrictions apply to the availability of these data. Since our study was supported by Korea Disease Control and Prevention Agency (KDCA), permission from KDCA is required to assess data.
